# Burden of invasive pneumococcal disease, non-invasive all-cause pneumonia, and acute otitis media in hospitalized US children: a retrospective multi-center study from 2015 to 2020

**DOI:** 10.1186/s12913-024-11898-w

**Published:** 2024-12-18

**Authors:** Salini Mohanty, Nicole Cossrow, Meghan White, Kalvin C. Yu, Gang Ye, Kristen A. Feemster, Vikas Gupta

**Affiliations:** 1 Outcomes Research, Merck & Co., Inc, 126 East Lincoln Ave., P.O. Box 2000, Rahway, NJ 07065 USA; 2 Becton, Dickinson & Company, Franklin Lakes, NJ USA; 3 Global and Medical Scientific Affairs, Merck & Co., Inc, Rahway, NJ USA

**Keywords:** Pneumococcal disease, *Streptococcus pneumoniae*, Costs, Hospitalization, Children, Antimicrobial resistance

## Abstract

**Background:**

Despite effective pneumococcal vaccines, pneumococcal disease (PD) exerts a substantial burden on children. This study explored the clinical and economic burden of invasive PD (IPD), non-invasive all-cause pneumonia (ACP), and acute otitis media (AOM) in hospitalized children.

**Methods:**

Data from the BD Insights Research Database of hospitalized children (< 18 years, including infants and children) in the US were analyzed retrospectively. The study cohort included patients with an ICD10 code for IPD, ACP, or AOM and/or a positive culture for *S. pneumoniae*. Descriptive statistics and multivariable analyses evaluated the following outcomes: length of stay [LOS], hospital cost per admission, hospital margin per admission [costs – payments], and in-hospital mortality.

**Results:**

The study included 4575 pediatric patients with IPD (*n* = 36), ACP (*n* = 3,329), or AOM (*n* = 1,210) admitted to 57 US hospitals from October 2015 to February 2020. Approximately half (50.7%) were under 2 years of age. The in-hospital mortality rate was 0.6% (*n* = 28). The observed median (interquartile range) LOS was 4 (3, 5) days, cost per admission was $4,240 ($2,434, $8,311) US dollars, and hospital margin per admission was -$63 ($2,118, $2,025). LOS and costs were highly variable according to clinical characteristics and hospital variables. Key variables associated with poor outcomes were having a moderate- or high-risk condition (chronic or immunocompromising), intensive care unit admission, and prior 90-day admission.

**Conclusions:**

The burden of PD among hospitalized pediatric patients in the US remains substantial. Our study highlights the burden of PD among young children (< 2 years) and children with underlying medical conditions that put them at greater risk for PD. The results support the need for ongoing prevention efforts including vaccination and antimicrobial stewardship programs to reduce the burden of PD in children.

**Supplementary Information:**

The online version contains supplementary material available at 10.1186/s12913-024-11898-w.

## Background

Despite decreases in *Streptococcus pneumoniae* incidence following introduction of pneumococcal conjugate vaccines (PCVs), pneumococcal disease (PD) continues to impact pediatric populations in the US [1–4]. Most surveillance data, including the Centers for Disease Control and Prevention (CDC) Active Bacterial Core surveillance, focus on invasive PD (IPD), including bacteremia and meningitis, due to the greater severity of disease and availability of diagnostics for IPD [2, 3]. However, non-invasive PD, such as non-invasive pneumonia and acute otitis media (AOM) due to pneumococcal infections, constitute the majority of PD infections and exert a substantial burden on the healthcare system [5–7]. The burden of PD is further exacerbated by antimicrobial resistant (AMR) *S. pneumoniae* infections [8–13].

For hospitalized patients in the US, hospital costs are generally covered to some extent by federal (Medicare, Medicaid) or private insurance. Reimbursement rates to hospitals vary depending on the needs of the patient and type of insurance. Hospital costs encompass expenses for most medical personnel, medications, testing, and other facility-related expenses. In general, the major determinant of hospital cost is usually length of stay. The hospital margin is determined by the hospital costs minus the amount received from insurance or other payors.

The objective of this study was to evaluate the clinical and economic outcomes associated with IPD, non-invasive all-cause pneumonia (ACP), and AOM in hospitalized children, including length of stay (LOS), hospital cost per admission, hospital margins per admission (cost minus payments), and in-hospital mortality. We further evaluated AMR in children with *S. pneumoniae*-positive cultures.

### Patients and methods

#### Study design

This was a retrospective study of administrative data and medical records from the BD Insights Research Database (Becton, Dickinson and Company, Franklin Lakes, NJ), which includes small and large hospitals in urban and rural areas and provides geographical representation across the US (Table 1). The data in this database are collected by hospitals during routine clinical care and analyzed by BD to provide insights into various clinical issues, including rates of clinical conditions, hospital financial outcomes, and antibiotic susceptibility rates [13, 14]. The patient population consisted of hospitalized pediatric patients (< 18 years, including infants and children) admitted between October 2015 and February 2020. Patients with an ICD10 code for IPD, ACP, or AOM and/or a positive culture for *S. pneumoniae* (blood and cerebrospinal/neurologic fluid for IPD; respiratory or ear/nose/throat for ACP or AOM) were included. The category of “ear/nose/throat culture source” reflects varying nomenclatures used at participating hospitals in our database. Accordingly, we were unable to identify tympanocentesis or spontaneous otorrhea within this category. Microbiologic results likely to be associated with colonization (including environmental/surveillance specimens such as rectal or nasal swabs) or contamination were excluded by use of a previously described algorithm [15]. ICD10 codes were used to differentiate IPD, ACP, and AOM. Cases of IPD were defined as pneumococcal bacteremia/septicemia, pneumococcal meningitis, bacteremic pneumococcal pneumonia, and other specified conditions where *S. pneumoniae* is specified in the ICD10 code, cases of ACP were defined as non-invasive pneumonia of any etiology (viral, bacterial, etc.), and cases of AOM were defined as AOM of any etiology (Supplementary Table 1). ICD10 codes for IPD included codes that specified *S. pneumoniae* as the bacterial pathogen, while ICD10 codes without pathogen attribution were used for non-invasive ACP and AOM (Supplementary Table 1), as current methods to detect bacterial pathogens in non-invasive pediatric pneumonia and AOM are extremely limited.

**Table 1:** Hospital numbers by medical facility characteristics and geographic location

Characteristic	*N* (%)
Total	57 (100)
Bed size	
< 100	13 (22.8)
100–300	28 (49.1)
> 300	16 (28.1)
Location	
Urban	38 (66.7)
Rural	19 (33.3)
Teaching status	
Teaching	27 (47.4)
Non-teaching	30 (52.6)
US census region	
East North Central	10 (17.5)
East South Central	11 (19.3)
Middle Atlantic	1 (1.8)
Mountain	5 (8.8)
New England	0
Pacific	4 (7.0)
South Atlantic	6 (10.5)
West North Central	2 (3.5)
West South Central	18 (31.6)

Percentages may not total to 100 due to rounding

Microbiology, antimicrobial susceptibility, and general laboratory results were based on local facility laboratory reports. Antimicrobial susceptibility was categorized as susceptible or resistant (defined as an antimicrobial susceptibility report of intermediate or resistant based on the interpretive criteria applied by the local laboratory) [11]. Risk factors for PD were based on ICD10 codes representing risk factors listed in the CDC’s Advisory Committee on Immunization Practices (ACIP) pediatric pneumococcal immunization recommendations [16, 17] and categorized as low (no risk conditions for PD), moderate (chronic medical conditions), and high (immunocompromising conditions) (Supplementary Table 2). Descriptive data for each admission were evaluated for outcomes of interest, including LOS (days), hospital cost per admission (US dollars), hospital margins per admission (payments received by the hospital from all sources, including insurance and patient payments, minus costs incurred by the hospital (in US dollars), and in-hospital mortality. The study was approved and exempted from consent by the New England/WGC Institutional Review Board/Human Subjects Research Committee (Wellesley, Massachusetts) based upon use of a limited retrospective dataset for an epidemiology study and conducted in compliance with Health Insurance Portability and Accountability Act requirements.

### Statistical analyses

The observed in-hospital mortality distribution by patient demographic and clinical characteristics was reported as count and percent, and the other study outcomes (LOS, hospital cost per admission, and hospital margin per admission) were reported as median and interquartile (IQR) range due to non-normally distributed data (Table 2). To evaluate the association between each study outcome and the potentially influencing factors, we used generalized linear mixed models (GLMM) with patients nested in hospitals as random effect to account for within-cluster correlation of data. Specifically, the random intercept logistic regression model was used to assess the association between in-hospital mortality and patient demographics (age, sex), insurance status, clinical characteristics (prior 90-day hospitalization, ICD10-based risk classification for pneumococcal disease [low, moderate, high], disease status [IPD, non-invasive ACP/AOM], *S. pneumoniae* culture-positive status, intensive care unit [ICU] admission status, and facility characteristics (bed size, teaching status, urban/rural location, and geographic region based on US Census regions). GLMM with a negative binomial distribution and a log link function modeled LOS, and GLMM with gamma distribution and a log link function modeled total hospital cost per admission. Hospital margin per admission was assessed using GLMM with identity (normal) distribution. All analyses were conducted using SAS version 9.4 (SAS Institute, Cary, NC, USA).

**Table 2:** Characteristics and observed outcomes in pediatric patients hospitalized with IPD, non-invasive ACP, or AOM

Characteristic	Patients *n* (% of all patients)	In-hospital mortality *n* (%)	LOS, days Median (IQR)	Hospital cost, Median USD (IQR)	Hospital margin Median USD (IQR)
All patients	4,575 (100%)	28 (0.6%)	4 (3, 5)	$4,240 ($2,434, $8,311)	-$63 (-$2,118, $2,025)
Disease status					
Non-invasive ACP/AOM^a^	4,539 (99.2%)	28 (0.6%)	4 (3, 5)	$4,214 ($2,413, $8,219)	-$67 (-$2,117, $1,999)
IPD	36 (0.8%)	0	11 (5.5, 18.5)	$9,098 ($6,552, $28,544)	$1,671 (-$5,902, $9,963)
*S. pneumoniae* culture positive					
No or not tested	4,534 (99.1%)	28 (0.6%)	4 (3, 5)	$4,214 ($2,413, $8,191)	-$64 (-$2,116, $1,991)
Yes	41 (0.9%)	0	8 (4, 13)	$9,393 ($6,160, $20,457)	$999 (-$3,660, $7,097)
Sex					
Female	2,069 (45.2%)	12 (0.6%)	4 (3, 5)	$4,261 ($2,460, $8,271)	-$133 (-$2,172, $1,952)
Male	2,506 (54.8%)	16 (0.6%)	4 (3, 5)	$4,211 ($2,413, $8,349)	-$5 (-$2,077, $2,068)
Age group					
< 2 years	2,321 (50.7%)	11 (0.5%)	4 (3, 5)	$3,930 ($2,283, $7,286)	$22 (-$1,712, $2,042)
2–4 years	1,033 (22.6%)	6 (0.6%)	4 (3, 5)	$3,859 ($2,189, $6,879)	$103 (-$1,643, $2,068)
5–17 years	1,221 (26.7%)	11 (0.9%)	4 (3, 7)	$5,555 ($3,028, $12,885)	-$469 (-$3,581, $1,938)
Prior 90-day admission					
No	3,930 (85.9%)	17 (0.4%)	4 (3, 5)	$4,064 ($2,368, $7,391)	-$1 (-$1,788, $2,042)
Yes	645 (14.1%)	11 (1.7%)	5 (3, 9)	$6,716 ($3,083, $18,753)	-$644 (-$5,848, $1,874)
ICU admission					
No	3,958 (86.5%)	12 (0.3%)	4 (3, 5)	$3,736 ($2,224, $6,391)	$103 (-$1,524, $2,019)
Yes	617 (13.5%)	16 (2.6%)	8 (5, 14)	$18,771 ($10,125, $39,141)	-$4,305 (-$14,208, $2,064)
Risk of PD					
Low risk (no risk factors)^b^	2,672 (58.4%)	4 (0.2%)	4 (3, 5)	$3,446 ($2,041, $6,247)	$333 (-$1,310, $2,094)
Moderate risk^c^	1,489 (32.6%)	18 (1.2%)	4 (3, 7)	$5,778 ($3,368, $12,582)	-$670 (-$3,796, $1,723)
High risk^d^	414 (9.0%)	6 (1.5%)	5 (3, 10)	$6,313 ($3,323, $20,020)	-$477 (-$5,018, $2,670)
Insurance					
Medicaid	2,891 (63.2%)	18 (0.6%)	4 (3, 6)	$4,188 ($2,396, $8,449)	-$594 (-$2,701, $857)
Private	1,413 (30.9%)	9 (0.6%)	4 (3, 5)	$4,362 ($2,480, $8,371)	$2,443 (-$96, $4,886)
Other^e^	201 (4.4%)	1 (0.5%)	4 (3, 5)	$4,424 ($2,744, $7,173)	-$710 (-$2,697, $981)
Uninsured	70 (1.5%)	0	3 (2, 4)	$3,256 ($1,693, $6,289)	-$2,924 (-$5,000, -$836)

Values are presented as *n* (%) unless otherwise indicated. Percentages within categories may not total 100 due to rounding

*ACP* non-invasive all-cause pneumonia, *AOM* acute otitis media, *ICU* intensive care unit, *IPD* invasive pneumococcal disease, *IQR* interquartile range, *LOS* length of stay, *PD* pneumococcal disease

^a^ACP, *n* = 3,329; AOM, *n* = 1,210

^b^None of the listed PD risk factors for moderate or high risk (see Supplementary Table 2 for specific ICD codes)

^c^Alcoholism, asthma, chronic heart disease, chronic liver disease, chronic lung disease, diabetes mellitus, and tobacco use (see Supplementary Table 2 for specific ICD codes)

^d^Asplenia, cancer, chronic renal disease, cerebrospinal fluid leak, cochlear implant, human immunodeficiency virus infection, immunocompromising diseases, and organ transplantation (see Supplementary Table 2 for specific ICD codes)

^e^Insurance types not encompassed in the other categories including Veterans’ Affairs and Tricare

## Results

The study included 4,575 pediatric patients with IPD, ACP, or AOM at 57 hospitals in the US. The hospitals included in this study were similar to the overall US distribution as reported by the American Hospital Association with respect to urban location (66.7% for this study vs. 61.2% for the US) but included a higher proportion of teaching hospitals (47.4% vs. 36.5%) (Table 1) [13].

Characteristics of the hospitalized pediatric patients with IPD, ACP, or AOM are shown in Table 2. In this patient cohort, 45.2% were female and 50.7% were under 2 years of age; the mean age (standard deviation [SD]) was 3.4 (4.4) years and the median age (IQR) was 1 (0, 5). Almost all patients (4,539 [99.2%]) had non-invasive disease; 36 (0.8%) had IPD. The majority of pediatric patients in this study had non-invasive ACP (3,329/4,575; 72.8%). A total of 1,210 (26.4%) patients had AOM. For purposes of these analyses, ACP and AOM were grouped together to allow evaluation of non-invasive disease.

A total of 4,534 patients (99.1%) were included in the study cohort based on meeting ICD10 code criteria only. A culture that was positive for *S. pneumoniae* was recorded for 41 (0.9%) patients. Of patients with a positive *S. pneumoniae* culture, 23/41 (56.1%) had isolates with resistance to at least one antibiotic class (penicillin, macrolides, fluoroquinolones, extended-spectrum cephalosporins, or tetracyclines).

### Unadjusted outcomes

The in-hospital mortality rate was 0.6% in this patient cohort (*n* = 28). All 28 mortality cases were in patients with non-invasive disease and in patients who were culture-negative or not tested for *S. pneumoniae*. Sixteen of the 28 mortality cases were admitted to the ICU (Table 2).

Because mean values for LOS and economic outcomes were associated with large SDs, median values are reported (Table 2). The median (IQR) LOS was 4 (3, 5) days per admission, the median cost was $4,240 ($2,434, $8,311) per admission, and the median margin was a hospital loss (negative margin) of -$63 (-$2,118, $2,025) per admission.

### Adjusted mortality outcomes

Mortality occurred in < 1% of admissions and therefore variables identified as related to mortality should be considered with caution. In adjusted analyses, factors significantly associated with in-hospital mortality were prior 90-day admission, ICU admission, and having a moderate- or high-risk condition for PD (Table 3). Patients’ age, sex, insurance type, and facility characteristics did not have a significant impact on mortality.

**Table 3:** Variables associated with in-hospital mortality in multivariable analyses of US children with IPD, ACP, or AOM (*N* = 4,575)

Characteristic	Odds ratio (95% CI)	*P* value
Prior 90-day admission		
No	ref	
Yes	2.59 (1.12, 5.98)	0.0256
ICU admission		
No	ref	
Yes	5.36 (2.38, 12.10)	< 0.0001
Risk of PD		
Low risk^a^	ref	
Moderate risk^b^	5.43 (1.79, 16.42)	0.0028
High risk^c^	5.84 (1.72, 19.86)	0.0047

*ACP* non-invasive all-cause pneumonia, *AOM* acute otitis media, *CI* confidence interval, *ICU* intensive care unit, IPD invasive pneumococcal disease, *PD* pneumococcal disease, *ref* reference

^a^None of the listed PD risk factors for moderate or high risk (see Supplementary Table 2 for specific ICD codes)

^b^Alcoholism, asthma, chronic heart disease, chronic liver disease, chronic lung disease, diabetes mellitus, and tobacco use (see Supplementary Table 2 for specific ICD codes)

^c^Asplenia, cancer, chronic renal disease, cerebrospinal fluid leak, cochlear implant, human immunodeficiency virus infection, immunocompromising diseases, and organ transplantation (see Supplementary Table 2 for specific ICD codes)

### Adjusted hospital LOS, cost per admission, and hospital margins

In the adjusted analyses (Table 4), the estimated LOS was a mean (SD) of 6.3 (5.5, 7.3) days and 12.7 (9.4, 17.1) days for non-invasive ACP/AOM and IPD, respectively, and hospital cost per admission was a mean (SD) of $15,756 ($13,207, $18,819) and $31,837 ($18,793, $53,934) for non-invasive ACP/AOM and IPD, respectively. Factors significantly associated with increased LOS were IPD, age < 2 years, prior 90-day admission, ICU admission, and having a moderate- or high-risk condition for PD (Table 4). Hospital costs per admission showed a similar pattern to LOS (Table 4). Factors significantly associated with higher hospital costs per admission were IPD, prior 90-day admission, ICU admission, and having a moderate- or high-risk condition for PD. The highest costs were observed in the age group of < 2 years, followed by the 5–17-year age group (reference group), which was significantly higher than the 2–4-year age group.

**Table 4:** Multivariate association of LOS, hospital costs, and hospital margins for US children with IPD, ACP, or AOM (*N* = 4,575)

Characteristic	Estimated LOS		Estimated hospital cost per admission		Estimated hospital margin per admission	
	Days	*P*	USD	*P*	USD	*P*
Disease status						
Non-invasive ACP/AOM	6.3 (5.5, 7.3)	< 0.0001	$15,765 ($13,207, $18,819)	0.0058	Not in model	
IPD	12.7 (9.4, 17.1)		$31,837 ($18,793, $53,934)			
Age group						
< 2 years	10.9 (8.9, 13.3)	< 0.0001	$26,429 ($19,279, $36,231)	0.4347	-$5,705 (-$8,176, -$3,235)	0.5693
2–4 years	7.7 (6.3, 9.5)	0.0562	$17,614 ($12,731, $24,372)	0.0028	-$4,637 (-$7,165, -$2,108)	0.0797
5–17 years	8.5 (6.9, 10.5)	ref	$24,154 ($17,114, $34,091)	ref	-$6,607 (-$9,409, -$3,805)	ref
Prior 90-day admission						
No	8.2 (6.8, 9.9)	0.0039	$18,193 ($13,479, $24,557)	0.0012	-$3,014 (-$4.672, -$1,356)	0.0038
Yes	9.7 (7.9, 12.0)		$27,587 ($19,330, $39,373)		-$8,285 (-$11,842, -$4,729)	
ICU admission						
No	6.3 (5.2, 7.6)	0.0039	$12,268 ($9,109, $16,523)	< 0.0001	Not in model	
Yes	12.7 (10.3, 15.8)		$40,912 ($29,207, $57,308)			
Risk of PD						
Low risk (no risk factors)^a^	6.5 (5.3, 7.9)	ref	$12,778 ($9,201, $17,746)	ref	-$3,298 (-$5,727, -$869)	ref
Moderate risk^b^	10.8 (8.8, 13.2)	< 0.0001	$32,125 ($23,704, $43,537)	< 0.0001	-$6,430 (-$9,162, -$3,697)	0.0589
High risk^c^	10.3 (8.1, 13.0)	< 0.0001	$27,392 ($18,914, $39,672)	< 0.0001	-$7,221 (-$11,646, -$2,797)	0.0937
Insurance						
Medicaid	9.1 (7.7, 10.7)	0.8256	$25,207 ($18,895, $33,629)	0.1833	-$7,773 (-$10,106, -$5,439)	< 0.0001
Private	9.0 (7.6, 10.7)	ref	$22,894 ($16,980, $30,867)	ref	$1,369 (-$1,423, $4,162)	ref
Other^d^	8.7 (6.9, 11.0)	0.7378	$21,831 ($15,168, $31,420)	0.6995	-$7,510 (-$10,258, -$4,763)	< 0.0001
Uninsured	9.0 (5.8, 13.9)	0.9823	$19,996 ($12,475, $32,051)	0.4909	-$8,685 (-$11,965, -$5,405)	< 0.0001

Data are presented as mean (95% CI) per admission. Analyses were adjusted for all variables shown in the table and for hospital urban/rural location, teaching status, and US census region (see Supplementary Table 3)

*ACP* non-invasive all-cause pneumonia, *AOM* acute otitis media, *CI* confidence interval, *ICU* intensive care unit, *IPD* invasive pneumococcal disease, *IPD* invasive pneumococcal disease, *LOS* length of stay, *PD* pneumococcal disease, *ref* reference, *USD* US dollars

^a^None of the listed PD risk factors for moderate or high risk (see Supplementary Table 2 for specific ICD codes)

^b^Alcoholism, asthma, chronic heart disease, chronic liver disease, chronic lung disease, diabetes mellitus, and tobacco use (see Supplementary Table 2 for specific ICD codes)

^c^Asplenia, cancer, chronic renal disease, cerebrospinal fluid leak, cochlear implant, human immunodeficiency virus infection, immunocompromising diseases, and organ transplantation (see Supplementary Table 2 for specific ICD codes)

^d^Insurance types not encompassed in the other categories including Veterans’ Affairs and Tricare

Patient variables significantly associated with higher hospital margins (greater financial loss per admission) were prior 90-day admission and having a moderate- or high-risk condition for PD. The estimated mean (SD) hospital margin per admission was -$3,298 (-$5,727, -$869) for children at low risk of PD, -$6,430 (-9,162, -$3,697) for children at moderate risk of PD, and -$7,221 (-$11,646, -$2,797) for children at high risk of PD. Non-private insurance was also associated with significantly higher negative margins per admission (Table 4).

Age showed a mixed pattern across these outcomes. Compared with the 5–17 year age group, children < 2 years of age had a longer LOS while the 2–4 year age group trended towards a significantly shorter LOS. Hospital costs per admission were also highest in the youngest age group, but not significantly different from costs in the 5–17 year reference age group, which had significantly higher costs than the 2–4 year age group. Negative margins (hospital loss) were highest in children 5–17 years of age, although the difference was not significantly different from the other age groups (Table 4).

Outcomes adjusted for hospital variables (urban/rural status, teaching status, and geographic location based on US census region) are shown in Supplementary Table 3. Urban location and teaching status were associated with longer LOS and higher hospital costs per admission, while the East South Central region had a significantly longer LOS, the Pacific region had significantly higher costs per admission, and the Middle Atlantic region had significantly greater negative hospital margins.

## Discussion

This study documented the clinical and economic burden associated with IPD, non-invasive ACP, and AOM in hospitalized children in the US, specifically an observed in-hospital mortality rate of 0.6%, median LOS of 4 days, median hospital cost of $4,240 per admission, and median hospital loss (negative margin) of $63 per admission. These findings are particularly notable given that the patient cohort primarily included non-invasive ACP and AOM, which is typically less severe than IPD but far more prevalent [1].

There were 28 patients with an outcome of in-hospital mortality. Risk factors for in-hospital mortality were prior 90-day admission, ICU admission, and having a condition of moderate or high risk for PD. These data indicate the critical importance of PD prevention efforts, particularly in patients with factors that put them at greater risk for PD, including chronic and immunocompromising conditions.

Patient-related variables associated with longer LOS, higher hospital costs per admission, and greater negative hospital margins per admission included IPD, prior 90-day admission, ICU admission, and having risk conditions that put them at greater risk for PD. Other studies have also identified associations between underlying comorbid conditions and high costs in patients with PD [18]. Younger age (< 2 years) was associated with a longer LOS and higher hospital costs per admission, which is consistent with a previous report of children with pneumonia [19], but the 5–17 year age group had the highest negative margins (hospital loss). Possible reasons behind the high costs and negative margins (hospital loss) in the 5–17 year age group remain unclear. One study found that children with comorbidities were older at the time of IPD diagnosis [20], providing a possible explanation for higher costs in this age group. Additional studies will be required to explore this potential association. Other variables that impacted economic outcomes were type of insurance and characteristics of the hospital including urban location, teaching status, and US census region. In particular, non-private insurance was associated with higher negative margins, suggesting that Medicaid and other non-private insurance programs provide less payment for PD than private insurers.

This study differs from most other reports on PD in the US pediatric population by reporting hospital costs per admission, rather than costs estimated from Markov models of simulated clinical events [19, 21–24]. These differences in study design make comparisons among studies difficult. The most closely comparable study is a recent study by Hu and colleagues in US children < 18 years based on administrative claims data which reported payments rather than costs [25]. In children with non-invasive ACP, Hu et al. [25] reported mean payments of $15,041 per inpatient admission for the Medicaid-insured children and $34,653 for the commercially insured children compared with estimated mean hospital costs of $15,765 for non-invasive ACP/AOM and $31,837 for IPD in the study reported here. An earlier study using 2008 to 2014 data from a commercial claims database reported a cost of $10,963 per hospitalized pneumonia episode for patients of all ages (including 31% of cases in patients < 18 years of age) but did not analyze pediatric cost data separately [19]. These three studies show the high costs associated with PD in children, including those with non-invasive PD. Our study extends these data by showing that, on average, hospitals lose an adjusted $3,298 for each case of PD in children with no underlying risk factors and up to $7,221 per case in immunocompromised children (high risk PD category). To the best of our knowledge, our study is the first to report on the hospital margins associated with the treatment of pediatric PD in US hospitals, thereby providing additional evidence of the financial impact of PD on the healthcare systems.

Blood and respiratory cultures are infrequently performed in children with ACP. In one study from the Pediatric Health Information System Plus database, 34% of children hospitalized with ACP had a blood culture performed and 2.5% had a positive culture, primarily *S. pneumoniae* [26]. Although in our study positive lab cultures were reported for only 41 patients, over half of these patients (*n* = 23) had antibiotic-resistant *S. pneumoniae*. This finding is consistent with other reports of high rates of AMR in *S. pneumoniae* isolates from pediatric and adult patients in the US [2, 11, 12, 27, 28]; increases in resistance are of particular concern for non-vaccine serotypes [10]. Although AMR status was not a significant predictor of outcomes in multivariable analyses, the number of AMR patients included in these analyses may have been too few to detect a significant association.

The high clinical and economic burden and AMR rates associated with PD in children support the need for expanded pneumococcal vaccination efforts. The majority of PD cases in children are caused by non-vaccine serotypes [29–31] and this trend is increasing in some locations [30], but not others [31]. In a prospective study examining IPD in children at 8 children’s hospitals in the US from 2014 to 2017, approximately three-quarters (76.1%) of IPD cases were caused by non-PCV13 *S. pneumoniae* serotypes and these serotypes are more common in patients with underlying risk factors [29], a variable identified here as a key risk factor for poor outcomes. Models suggest that the introduction of effective vaccines protecting against additional *S. pneumoniae* serotypes may play a key role in reducing not only overall disease burden [7, 21, 32–34], but also AMR *S. pneumoniae* infections [10]. In addition to higher valency vaccines, more widespread vaccination may also diminish PD impact in children. Pneumococcal vaccination rates in US children are generally high, but some populations, including uninsured children and children living below the poverty level, have suboptimal coverage [35]. Our data also support the importance of protection in older children with risk factors for PD. Targeted vaccination campaigns may help address these at-risk populations.

Study limitations include low representation of pediatric patients from certain geographic regions and a small number of patients with culture results. Although we used an algorithm to identify potential contaminants [15], it is possible that not all contaminant culture results were excluded. Selection bias may have affected AMR rates, as more severely ill patients are more likely to have cultures performed. AMR rates may also have been affected by variations in breakpoints and susceptibility tests used by different facilities. This study was intentionally designed to focus on ICD10 codes as a determinant of PD. Blood and respiratory cultures are not routinely performed in children with suspected pneumonia or PD and provide limited information concerning a causative pathogen [26, 36]. This study did not evaluate *S. pneumoniae* serotypes or vaccination status. Future studies stratifying outcomes by vaccination status are warranted. Future studies should also endeavor to evaluate specific age groups within the pediatric population. We did not have access to causes of death or the reasons why some mortality cases were not admitted to the ICU. The study period did not span the COVID-19 pandemic period; rates of IPD [37] and AMR [38] were both affected by pandemic-related factors. The AOM episodes in our study of hospitalized patients likely reflected complicated AOM, which is less common than milder forms of AOM that are typically treated in outpatient settings. In the US, AOM is a leading cause of physician office visits and antibiotic prescriptions in children and is primarily managed in outpatient settings [39], but it is also observed in inpatient settings. A study based on the Nationwide Emergency Department Sample database from 2009 to 2011 found that although 99.44% of 5,778,462 children seen in US emergency rooms for AOM were treated and released, 32,665 AOM patients were admitted [40]. This finding is consistent with our data, which included 1,210 pediatric patients with AOM. We therefore considered it important to include AOM cases to provide a comprehensive overview of the epidemiology of PD in hospitalized children.

In conclusion, this study documents a substantial burden of IPD, non-invasive ACP, and AOM, particularly non-invasive ACP, in hospitalized children in the US. Populations at high risk for poor outcomes include young children (< 2 years) and those with risk conditions that put them at greater risk for PD. The significant impact of pediatric IPD, ACP, and AOM on patients and health systems highlights the need for widespread prevention efforts including pneumococcal vaccination and antimicrobial stewardship programs.

## Supplementary Information

Supplementary Material 1.

Supplementary Material 2.

Supplementary Material 3.

## Data Availability

The datasets developed and/or analyzed during the current project are available from the corresponding author on reasonable request.

## Abbreviations

ACIP: Advisory Committee on Immunization Practices
ACP: All-cause pneumonia (non-invasive)
AMR: Antimicrobial resistance
AOM: Acute otitis media
BD: Becton, Dickinson & Company
CDC: Centers for Disease Control and Prevention
CI: Confidence interval
GLMM: Generalized linear mixed models
ICU: Intensive care unit
IPD: Invasive pneumococcal disease
IQR: Interquartile range
LOS: Length of stay
PCV: Pneumococcal conjugate vaccine
PD: Pneumococcal disease
SD: Standard deviation
USD: United States dollars

## References

[1] Wahl B, O’Brien KL, Greenbaum A, et al. Burden of *Streptococcus pneumoniae* and *Haemophilus influenzae* type b disease in children in the era of conjugate vaccines: global, regional, and national estimates for 2000-15. Lancet Glob Health. 2018;6(7):e744–57. 10.1016/s2214-109x(18)30247-x. 29903376 10.1016/S2214-109X(18)30247-X PMC6005122

[2] Centers for Disease Control and Prevention. Active Bacterial Core Surveillance (ABCs) report. Emerging infections program network, Streptococcus pneumoniae, 2020. https://www.cdc.gov/abcs/downloads/SPN_Surveillance_Report_2020.pdf. Accessed 23 Jan 2023.

[3] Wasserman M, Chapman R, Lapidot R, et al. Twenty-year public health impact of 7- and 13-valent pneumococcal conjugate vaccines in US children. Emerg Infect Dis. 2021;27(6):1627–36. 10.3201/eid2706.204238. 34013855 10.3201/eid2706.204238 PMC8153862

[4] Hu T, Song Y, Done N, et al. Incidence of invasive pneumococcal disease in children with commercial insurance or Medicaid coverage in the United States before and after the introduction of 7- and 13-valent pneumococcal conjugate vaccines during 1998–2018. BMC Public Health. 2022;22(1):1677. 10.1186/s12889-022-14051-6. 36064378 10.1186/s12889-022-14051-6 PMC9442936

[5] Tong S, Amand C, Kieffer A, Kyaw MH. Trends in healthcare utilization and costs associated with acute otitis media in the United States during 2008–2014. BMC Health Serv Res. 2018;18(1):318. 10.1186/s12913-018-3139-1. 29720156 10.1186/s12913-018-3139-1 PMC5932897

[6] Florin TA, Byczkowski T, Gerber JS, Ruddy R, Kupperman N. Diagnostic testing and antibiotic use in young children with community-acquired pneumonia in the United States, 2008–2015. J Pediatr Infect Dis Soc. 2020;9(2):248–52. 10.1093/jpids/piz026. 10.1093/jpids/piz026 PMC7192397 31107533

[7] Wasserman MD, Perdrizet J, Grant L, et al. Clinical and economic burden of pneumococcal disease due to serotypes contained in current and investigational pneumococcal conjugate vaccines in children under five years of age. Infect Dis Ther. 2021;10(4):2701–20. 10.1007/s40121-021-00544-1. 34633639 10.1007/s40121-021-00544-1 PMC8503717

[8] Gaviria-Agudelo CL, Jordan-Villegas A, Garcia C, McCracken GH Jr. The effect of 13-valent pneumococcal conjugate vaccine on the serotype distribution and antibiotic resistance profiles in children with invasive pneumococcal disease. J Pediatr Infect Dis Soc. 2017;6(3):253–9. 10.1093/jpids/piw005. 10.1093/jpids/piw005 PMC7107452 26907814

[9] Andrejko K, Ratnasiri B, Hausdorff WP, Laxminarayan R, Lewnard JA. Antimicrobial resistance in paediatric *Streptococcus pneumoniae* isolates amid global implementation of pneumococcal conjugate vaccines: a systematic review and meta-regression analysis. Lancet Microbe. 2021;2(9):e450–60. 10.1016/s2666-5247(21)00064-1. 34485957 10.1016/S2666-5247(21)00064-1 PMC8410609

[10] Bajema KL, Gierke R, Farley MM, et al. Impact of pneumococcal conjugate vaccines on antibiotic-nonsusceptible invasive pneumococcal disease in the United States. J Infect Dis. 2022;226(2):342–51. 10.1093/infdis/jiac154. 35481950 10.1093/infdis/jiac154

[11] Mohanty S, Feemster K, Yu KC, Watts JA, Gupta V. Trends in *Streptococcus pneumoniae* antimicrobial resistance trends in US children: a US multicenter evaluation. Open Forum Infect Dis. 2023;10(3):ofad098. 10.1093/ofid/ofad098. 36968964 10.1093/ofid/ofad098 PMC10034583

[12] Kaur R, Pham M, Yu KOA, Pichichero ME. Rising pneumococcal antibiotic resistance in the post-13-valent pneumococcal conjugate vaccine era in pediatric isolates from a primary care setting. Clin Infect Dis. 2021;72(5):797–805. 10.1093/cid/ciaa157. 32067037 10.1093/cid/ciaa157 PMC7935395

[13] Centers for Disease Control and Prevention. Antibiotic resistance threats in the United States, 2019. https://www.cdc.gov/DrugResistance/Biggest-Threats.html. Accessed 7 Mar 2024.

[14] Mohanty S, Cossrow N, Yu KC, Ye G, White M, Gupta V. Clinical and economic burden of invasive pneumococcal disease and noninvasive all-cause pneumonia in hospitalized US adults: a multicenter analysis from 2015 to 2020. Int J Infect Dis. 2024;143: 107023. 10.1016/j.ijid.2024.107023. 38555060 10.1016/j.ijid.2024.107023

[15] Brossette SE, Hacek DM, Gavin PJ, et al. A laboratory-based, hospital-wide, electronic marker for nosocomial infection: the future of infection control surveillance? Am J Clin Pathol. 2006;125(1):34–9. 16482989

[16] Kobayashi M, Farrar JL, Gierke R, et al. Use of 15-valent pneumococcal conjugate vaccine among U.S. children: updated recommendations of the Advisory Committee on Immunization practices–United States, 2022. Morb Mortal Wkly Rep. 2022;71(37):1174–81. 10.15585/mmwr.mm7137a3. 10.15585/mmwr.mm7137a3 PMC9484809 36107786

[17] Healthcare Cost and Utilization Project. User guide: Elixhauser comorbidity software refined for ICD-10-CM diagnoses, v2021.1. 2020. https://hcup-us.ahrq.gov/toolssoftware/comorbidityicd10/Com-ICD10CM-User-Guide-v2021-1.pdf. Accessed 23 Jan 2024.

[18] Weycker D, Farkouh RA, Strutton DR, Edelsberg J, Shea KM, Pelton SI. Rates and costs of invasive pneumococcal disease and pneumonia in persons with underlying medical conditions. BMC Health Serv Res. 2016;16:182. 10.1186/s12913-016-1432-4. 27177430 10.1186/s12913-016-1432-4 PMC4867996

[19] Tong S, Amand C, Kieffer A, Kyaw MH. Trends in healthcare utilization and costs associated with pneumonia in the United States during 2008–2014. BMC Health Serv Res. 2018;18(1):715. 10.1186/s12913-018-3529-4. 30217156 10.1186/s12913-018-3529-4 PMC6137867

[20] Yildirim I, Shea KM, Little BA, Silverio AL, Pelton SI, Members of the Massachusetts Department of Public Health. Vaccination, underlying comorbidities, and risk of invasive pneumococcal disease. Pediatrics. 2015;135(3):495–503. 10.1542/peds.2014-2426. 25647674 10.1542/peds.2014-2426

[21] Hu T, Weiss T, Owusu-Edusei K, Petigara T. Health and economic burden associated with 15-valent pneumococcal conjugate vaccine serotypes in children in the United States. J Med Econ. 2020;23(12):1653–60. 10.1080/13696998.2020.1840216. 33084447 10.1080/13696998.2020.1840216

[22] Rubin JL, McGarry LJ, Strutton DR, et al. Public health and economic impact of the 13-valent pneumococcal conjugate vaccine (PCV13) in the United States. Vaccine. 2010;28(48):7634–43. 10.1016/j.vaccine.2010.09.049. 20883739 10.1016/j.vaccine.2010.09.049

[23] Ray GT, Pelton SI, Klugman KP, Strutton DR, Moore MR. Cost-effectiveness of pneumococcal conjugate vaccine: an update after 7 years of use in the United States. Vaccine. 2009;27(47):6483–94. 10.1016/j.vaccine.2009.08.045. 19720366 10.1016/j.vaccine.2009.08.045

[24] Huang M, Hu T, Weaver J, Odwusu-Edusei K, Elbasha E. Cost-effectiveness analysis of routine use of 15-valent pneumococcal conjugate vaccine in the US pediatric population. Vaccines (Basel). 2023;11(1): 135. 10.3390/vaccines11010135. 36679980 10.3390/vaccines11010135 PMC9861214

[25] Hu T, Song Y, Done N, et al. Economic burden of acute otitis media, pneumonia, and invasive pneumococcal disease in children in the United States after the introduction of 13-valent pneumococcal conjugate vaccines during 2014–2018. BMC Health Serv Res. 2023;23(1):398. 10.1186/s12913-023-09244-7. 37098521 10.1186/s12913-023-09244-7 PMC10127426

[26] Neuman MI, Hall M, Lipsett SC, et al. Pediatric research in inpatient settings network. Utility of blood culture among children hospitalized with community-acquired pneumonia. Pediatrics. 2017;140(3):e20171013. 10.1542/peds.2017-1013. 28835382 10.1542/peds.2017-1013 PMC5574722

[27] Suaya JA, Mendes RE, Sings HL, et al. *Streptococcus pneumoniae* serotype distribution and antimicrobial nonsusceptibility trends among adults with pneumonia in the United States, 2009–2017. J Infect. 2020;81(4):557–66. 10.1016/j.jinf.2020.07.035. 32739491 10.1016/j.jinf.2020.07.035

[28] Mohanty S, Johnson KD, Yu KC, et al. A multicenter evaluation of trends in antimicrobial resistance among *Streptococcus pneumoniae* isolates from adults in the United States. Open Forum Infect Dis. 2022;9(9):ofac420. 10.1093/ofid/ofac420. 36168549 10.1093/ofid/ofac420 PMC9511122

[29] Kaplan SL, Barson WJ, Lin PL, et al. Invasive pneumococcal disease in children’s hospitals: 2014–2017. Pediatrics. 2019;144(3):e20190567. 10.1542/peds.2019-0567. 31420369 10.1542/peds.2019-0567

[30] Grewal R, Hillier K, Deeks SL, et al. Invasive pneumococcal disease epidemiology and serotype replacement after the introduction of the 13-valent pneumococcal conjugate vaccine in Ontario, Canada, 2007–2022. Open Forum Infect Dis. 2024;11(6):ofae275. 10.1093/ofid/ofae275. 38868312 10.1093/ofid/ofae275 PMC11167672

[31] Bertran M, D’Aeth JC, Abdullahi F, Eletu S, Andrews NJ, Ramsay ME, et al. Invasive pneumococcal disease 3 years after introduction of a reduced 1 + 1 infant 13-valent pneumococcal conjugate vaccine immunisation schedule in England: a prospective national observational surveillance study. Lancet Infect Dis. 2024;24(5):546–56. 10.1016/S1473-3099(23)00706-5. 38310905 10.1016/S1473-3099(23)00706-5

[32] Grant LR, Slack MPE, Theilacker C, et al. Distribution of serotypes causing invasive pneumococcal disease in children from high-income countries and the impact of pediatric pneumococcal vaccination. Clin Infect Dis. 2023;76(3):e1062-70. 10.1093/cid/ciac475. 35789262 10.1093/cid/ciac475 PMC9907512

[33] Huang L, Wasserman M, Grant L, et al. Burden of pneumococcal disease due to serotypes covered by the 13-valent and new higher-valent pneumococcal conjugate vaccines in the United States. Vaccine. 2022;40(33):4700–8. 10.1016/j.vaccine.2022.06.024. 35753839 10.1016/j.vaccine.2022.06.024

[34] Mohanty S, Hu T, Yang G, Khan TK, Owusu-Edusei K, Sukarom I. Health and economic burden associated with 15-valent pneumococcal conjugate vaccine serotypes in Korea and Hong Kong. Hum Vaccin Immunother. 2022;18(5): e2046433. 10.1080/21645515.2022.2046433. 10.1080/21645515.2022.2046433 PMC9196648 35420975

[35] Hill HA, Chen M, Elam-Evans LD, Yankey D, Singleton JA. Vaccination coverage by age 24 months among children born during 2018–2019—National Immunization Survey-Child, United States, 2019–2021. MMWR Morb Mortal Wkly Rep. 2023;72(2):33–8. 10.15585/mmwr.mm7202a3. 36634013 10.15585/mmwr.mm7202a3 PMC9869730

[36] Green A, Cockroft JL, Kaufman RA, McCullers JA, Arnold SR. Utility of induced sputum in assessing bacterial etiology for community-acquired pneumonia in hospitalized children. J Pediatr Infect Dis Soc. 2022;11(6):274–82. 10.1093/jpids/piac014. 10.1093/jpids/piac014 35363300

[37] Bertran M, Amin-Chowdhury Z, Sheppard CL, et al. Increased incidence of invasive pneumococcal disease among children after COVID-19 pandemic, England. Emerg Infect Dis. 2022;28(8):1669–72. 10.3201/eid2808.220304. 35876698 10.3201/eid2808.220304 PMC9328924

[38] Bauer KA, Puzniak LA, Yu KC, et al. A multicenter comparison of prevalence and predictors of antimicrobial resistance in hospitalized patients before and during the severe acute respiratory coronavirus 2 pandemic. Open Forum Infect Dis. 2022;9(11): ofac537. 10.1093/ofid/ofac537. 36381612 10.1093/ofid/ofac537 PMC9619740

[39] Talathi S, Gupta N, Sethuram S, Khanna S, Sitnitskaya Y. Otitis media in fully vaccinated preschool children in the pneumococcal conjugate vaccine era. Glob Pediatr Health. 2017;4: 2333794X17749668. 10.1177/2333794x17749668. 29308427 10.1177/2333794X17749668 PMC5751904

[40] Ren Y, Sethi RKV, Stankovic KM. Acute otitis media and associated complications in United States emergency departments. Otol Neurotol. 2018;39(8):1005–11. 10.1097/MAO.0000000000001929. 30113560 10.1097/MAO.0000000000001929 PMC6097248

