# Supplementary material for: Burden of invasive pneumococcal disease, non-invasive all-cause pneumonia, and acute otitis media in hospitalized US children: a retrospective multi-center study from 2015 to 2020
Source: BMC Health Serv Res. 2024 Dec 18;24:1574. doi: 10.1186/s12913-024-11898-w (PMC11653741; doi:10.1186/s12913-024-11898-w)
Supplement: Supplementary file 3 — Supplementary Material 3. [file 12913_2024_11898_MOESM3_ESM.docx]

**Supplementary Table 3** Hospital characteristics associated with LOS, hospital costs, and hospital margins for US children with IPD, ACP, or AOM (N=4,575).

| **Characteristic** | **Estimated LOS** | | **Estimated hospital cost per admission** | | **Estimated hospital margin per admission** | |
| --- | --- | --- | --- | --- | --- | --- |
|  | **Days** | **P** | **USD** | **P** | **USD** | **P** |
| Hospital location |  |  |  |  | Not in model^a^ | |
| Rural | 8.3 (6.8, 10.2) | 0.0129 | $19,819  ($14,072, $27,914) | 0.0382 |  |  |
| Urban | 9.6 (7.9, 11.7) |  | $25,325  ($18,627, $34,429) |  |  |  |
| Teaching status |  |  |  |  | Not in model^a^ | |
| Non-teaching | 7.6 (6.2, 9.4) | <0.0001 | $17,377  ($12,548, $24,066) | <0.0001 |  |  |
| Teaching | 10.5 (8.7, 12.7) |  | $28,883  ($21,272, $39,217) |  |  |  |
| US census region |  |  |  |  |  |  |
| East North Central | 7.1 (5.3, 9.6) | 0.0271 | $20,419  ($13,945, $29,900) | 0.7349 | -$7,116  (-$12,542, -$1,690) | 0.3460 |
| East South Central | 11.1 (9.1, 13.5) | 0.0032 | $19,908  ($14,180, $27,949) | 0.8477 | -$5,203  (-$7,657, -$2,750) | 0.4383 |
| Middle Atlantic | 6.6 (5.3, 8.3) | <0.0001 | $21,698  ($14,903, $31,591) | 0.4537 | -$11,746  (-$14,525, -$8,996) | <0.0001 |
| Mountain | 10.0 (8.1, 12.4) | 0.1843 | $23,011  ($16,226, $32,635) | 0.2208 | -$2,206  (-$5,218, $806) | 0.0919 |
| Pacific | 10.3 (7.5, 14.2) | 0.3329 | $42,485  ($27,320, $66,068) | <0.0001 | -$5,179  (-$9,224, -$1,135) | 0.6778 |
| South Atlantic | 8.9 (7.1, 11.0) | 0.8698 | $17,136  ($12,317, $23,840) | 0.3184 | -$4,609  (-$7,033, -$2,185) | 0.8624 |
| West North Central | 9.0 (7.3, 11.1) | ref | $19,370  ($13,753, $27,281) | ref | -$4,434  (-$6,783, -$2,086) | Ref |
| West South Central | 9.7 (8.0, 11.7) | 0.3358 | $22,172  ($16,025, $30,678) | 0.3457 | -$4,704  (-$7,534, -$1,873) | 0.8374 |

Data are presented as mean (95% CI) per admission. Analyses were adjusted for variables shown in this table and in Table 3.

ACP, non-invasive all-cause pneumonia; AOM, acute otitis media; CI, confidence interval; IPD, invasive pneumococcal disease; LOS, length of stay; USD, US dollars

^a^ Not included in the final model for this outcome due to statistical insignificance and/or not improving model fit
